# Influence of loop diuretics on denosumab-induced hypocalcaemia in osteoporosis: a retrospective observational analysis

**DOI:** 10.1186/s40780-024-00380-8

**Published:** 2024-09-27

**Authors:** Toshinori Hirai, Yukari Mori, Toru Ogura, Yuki Kondo, Yuka Sakazaki, Yoichi Ishitsuka, Akihiro Sudo, Takuya Iwamoto

**Affiliations:** 1 Department of Pharmacy, Faculty of Medicine, Mie University Hospital, Mie University, 2-174 Edobashi, Tsu, Mie 514-8507 Japan; 2 Clinical Research Support Center, Mie University Hospital, 2-174 Edobashi, Tsu, Mie 514-8507 Japan; 3 Department of Clinical Chemistry and Informatics, Graduate School of Pharmaceutical Sciences, Kumamoto University, 5-1 Oehonmachi, Chuo-ku, Kumamoto, Kumamoto, 862-0973 Japan; 4 Department of Orthopaedic Surgery, Mie University Graduate School of Medicine, 2-174 Edobashi, Tsu, Mie 514-8507 Japan; 5 Department of Pharmacy, Tokyo Medical and Dental University Hospital, Tokyo Medical and Dental University (TMDU), 1-5-45 Yushima, Bunkyo-ku, Tokyo 113-8519 Japan

**Keywords:** Denosumab, Loop diuretics, Hypocalcaemia, Calcium

## Abstract

**Background:**

We examined whether denosumab-induced hypocalcaemia is evident in osteoporosis when given loop diuretics that promote urinary calcium excretion.

**Methods:**

Japanese Spontaneous Adverse Drug Event Reports was analyzed to examine signals for denosumab-induced hypocalcaemia co-administered loop diuretics. We retrospectively included osteoporotic patients to detect predictors for denosumab-induced hypocalcaemia (corrected calcium level < 8.5 mg/dL) using multivariate logistic regression analysis. We compared differences in corrected calcium levels (ΔCa = nadir-baseline).

**Results:**

A significant signal for hypocalcaemia was detected (Reporting odds ratio = 865.8, 95% confidence interval [95% CI]: 596.8 to 1255.9, *p* < 0.0001). Among 164 patients (hypocalcaemia, 12%), loop diuretics have a significant association with hypocalcaemia (odds ratio [OR] = 6.410, 95% CI: 1.005 to 40.90, *p* = 0.0494). However, hypocalcaemia was found to be lower in high corrected calcium levels at baseline (OR = 0.032, 95% CI: 0.005 to 0.209, *p* < 0.0001) and calcium and vitamin D supplementation (OR = 0.285, 95% CI: 0.094 to 0.868, *p* = 0.0270). In the non-hypocalcaemia, ΔCa decreased significantly in the denosumab plus loop diuretics than in the denosumab alone (-0.9 [-1.3 to -0.7] mg/dL vs. -0.5 [-0.8 to -0.3] mg/dL, *p* = 0.0156). However, ΔCa remained comparable in the hypocalcaemia despite loop diuretics co-administration (-1.0 [-1.2 to -0.8] mg/dL vs. -0.8 [-1.5 to -0.7] mg/dL, *p* = 0.7904).

**Conclusions:**

Loop diuretics may predispose to developing denosumab-induced hypocalcaemia.

**Supplementary Information:**

The online version contains supplementary material available at 10.1186/s40780-024-00380-8.

## Background

Denosumab is a fully human monoclonal antibody that binds to the nuclear factor-kB ligand-receptor activator, inhibiting osteoclast action and bone resorption [1]. The randomized placebo-controlled trial showed a high efficacy profile for denosumab 60 mg administered subcutaneously every six months by increasing bone mineral density and decreasing the risk of vertebral, non-vertebral, and hip fractures in osteoporosis [2]. Because denosumab lowers osteoclast-mediated bone resorption significantly [3], hypocalcaemia remains a major concern in 7–26% of patients treated with denosumab [4–6]. In contrast, the prescribing information of denosumab highlights that the risk of hypocalcaemia is limited in female patients [7], which indicates a critically different risk of hypocalcaemia.

Previous studies have suggested that the risk of denosumab-induced hypocalcaemia is higher among patients with higher bone turnover and lower pre-treatment estimated glomerular filtration rate (eGFR) and serum calcium levels corrected for albumin [5, 8]. Although loop diuretics are treatment options to treat hypertension and/or edema, loop diuretics also frequently cause hypocalcaemia in patients, including those with osteoporosis [9, 10]. Nevertheless, little is known about the risk of denosumab-induced hypocalcaemia in patients receiving loop diuretics that promote calcium urinary excretion in a clinical setting.

Based on the pharmacological action of loop diuretics, we hypothesized that loop diuretics increase the risk of denosumab-induced hypocalcaemia. This study aimed to investigate the increased risk of denosumab-induced hypocalcaemia with the concomitant use of loop diuretics in patients with osteoporosis. To achieve this objective, we screened for the presence of drug-drug interactions using the Japanese spontaneous reporting database for adverse drug reactions. In addition, we validated the results obtained from the screening by utilizing an in-house medical dataset.

## Materials and methods

### Spontaneous adverse drug event reports analysis

The publicly available source, Japanese Adverse Drug Event Report database (JADER) database collects adverse event reports submitted to the Pharmaceutical and Medical Devices Agency. The JADER database accumulates cases of adverse drug reactions spontaneously reported by healthcare professionals and pharmaceutical companies. A screening of the signal of drug-drug interaction between denosumab and loop diuretics was carried out. We accessed the Pharmaceuticals and Medical Devices Agency website on 22 August 2023 and downloaded the dataset. The dataset includes “DEMO” (demographic information), “DRUG” (drug administration information), “REAC” (adverse event information), and “HIST” (comorbidity information). Hypocalcaemia was defined using the Japanese version of the Medical Dictionary for Regulatory Activities (MedDRA/J) ver. 25.1 J. We identified hypocalcaemia using three Preferred Terms (10,079,306/Neonatal hypocalcaemia, 10,020,947/Hypocalcaemia, 10,072,456/Hypocalcaemic seizure). To identify cases involving the use of denosumab for osteoporosis, cases where Pralia® was not mentioned in the brand name and the dose was not 60 mg (the approved dose of denosumab for osteoporosis in Japan) were excluded from the dataset. After eliminating duplicate records, we performed a disproportionality analysis using a 2 × 2 contingency table to calculate the reporting odds ratio (ROR) and 95% confidence interval (95% CI) as follows [11]:

$$ROR=\frac{a/c}{b/d}=\frac{ad}{bc}$$

$$95\%\ CI=exp\left[\text{log}\left(ROR\right)\pm 1.96\sqrt{\frac{1}{a}+\frac{1}{b}+\frac{1}{c}+\frac{1}{d}}\right]$$

where *a* represents target drugs with hypocalcaemia, *b* represents non-target drugs with hypocalcaemia, *c* represents target drugs without hypocalcaemia, and *d* represents non-target drugs without hypocalcaemia. We adopted the following criteria to define positive signals of drug-drug interactions: (1) at least three cases of hypocalcaemia in the group co-administered loop diuretics, (2) a lower limit of the 95% CI of the ROR in the group co-administered loop diuretics that exceeded 1.0, and (3) higher RORs in the group co-administered loop diuretics than in the other groups and mutually exclusive 95% CIs.

### Study design

The Clinical Research Ethics Review Committee of Mie University Hospital approved the study design, and the study complies with the Declaration of Helsinki and its later amendments (H2023-087). A retrospective observational cohort data included osteoporotic patients aged ≥ 18 years who received a 60 mg dose of denosumab (Pralia®, Daiichi Sankyo, Tokyo, Japan) subcutaneously every six months and underwent serum calcium tests at Mie University Hospital between June 2013 and September 2022. We excluded patients with hypocalcaemia (serum calcium level < 8.5 mg/dL) [12] at the initiation of denosumab (baseline) and those in whom serum calcium levels were not measured after denosumab administration. If the serum albumin level was < 4.0 g/dL (hypoalbuminemia), the serum calcium level was corrected using the following Equation [13].

$$\text{Corrected calcium}=\text{serum calcium}+\left(4.0-\text{serum albumin}\right)$$

Electronic medical charts were reviewed to construct a database of patients receiving denosumab. The collected data included demographic information, comorbidities, laboratory data, and co-administered medications (e.g., calcium and vitamin D supplements and loop diuretics) [14] during the period of denosumab administration. The kidney function was calculated using the following Equation [15]:

$$\text{eGFR}=194\times {\text{serum creatinine}}^{-1.094}\times {\text{age}}^{-0.287}\times 0.739\left(\text{if female}\right)$$

Furosemide equivalent dosage was calculated from the following conversion ratios [16, 17].

$$\text{Oral furosemide}:\text{azosemide}=20\text{ mg}:30\text{ mg}$$

$$\text{Oral furosemide}:\text{torasemide}=40\text{ mg}:10\text{ mg}$$

### Endpoint

The primary endpoint was hypocalcaemia (a corrected calcium level of < 8.5 mg/dL) [12]. All blood sampling was collected immediately near each denosumab administration. In cases where multiple measurements of hypocalcaemia were available, we selected the data point closest to the first administration of denosumab. The secondary endpoint was a difference in corrected calcium level (ΔCa, mg/dL) between baseline and nadir.

$$\triangle\mathrm{Ca}=\mathrm{serum}\;\mathrm{calcium}\;\mathrm{at}\;\mathrm{nadir}\;-\;\mathrm{serum}\;\mathrm{calcium}\;\mathrm{at}\;\mathrm{baseline}$$

### Statistical analysis

JMP® Pro 16.2.0 (SAS Institute Inc., Cary, NC, USA) was used for statistical analysis. All statistical tests were two-tailed, and a *p*-value < 0.05 was considered statistically significant. Continuous variables are presented as median [interquartile range] and compared using the Mann–Whitney U-test (unpaired) or Wilcoxon signed-rank test (paired). Categorical variables are presented as numbers (%). The chi-square test was used to evaluate the heterogeneity between groups.

Multivariate logistic regression analysis was employed to identify risk factors influencing hypocalcaemia using a forced entering of eGFR and a stepwise forward selection method with Akaike's Information Criterion (AIC), considering independent variables that exhibited a significance level of *p* < 0.20 in the univariate logistic regression analysis. The definition of multicollinearity was correlation efficient > 0.90. An independent variable was selected based on its clinical relevance in the case of multicollinearity between the independent variables. We confirmed the final model using a stepwise backward selection method. The final model outputted odds ratio (OR) and 95% CI. The interaction was further analyzed by introducing the terms of loop diuretics and independent variables.

Furthermore, we compared ΔCa between patients receiving denosumab plus loop diuretics (Dmab + LD) and receiving denosumab alone (Dmab alone) among patients with hypocalcaemia or non-hypocalcaemia.

## Results

### Spontaneous adverse drug event reports analysis

Screening of JADER database identified a drug-drug interaction signal for hypocalcaemia when co-administered with denosumab and loop diuretics (Table 1, ROR = 865.8, 95% CI: 596.8 to 1255.9, *p* < 0.0001).

**Table 1:** Disproportionality analysis for hypocalcaemia-induced denosumab when given loop diuretics

	**Non-hypocalcaemia**	**Hypocalcaemia**	**ROR**	**95% CI**	***P*** value
Denosumab	1346	141	148.6	122.6 to 180.0	< 0.0001
Loop diuretics	41,936	85	2.875	2.288 to 3.612	< 0.0001
Denosumab + loop diuretics	77	47	865.8	596.8 to 1255.9	< 0.0001
Non-target drugs	804,214	567	1.000	-	-

*Abbreviations*: *ROR* Reporting odds ratio, *95% CI*, 95% confidence interval

### Study participants

**Fig. 1:**
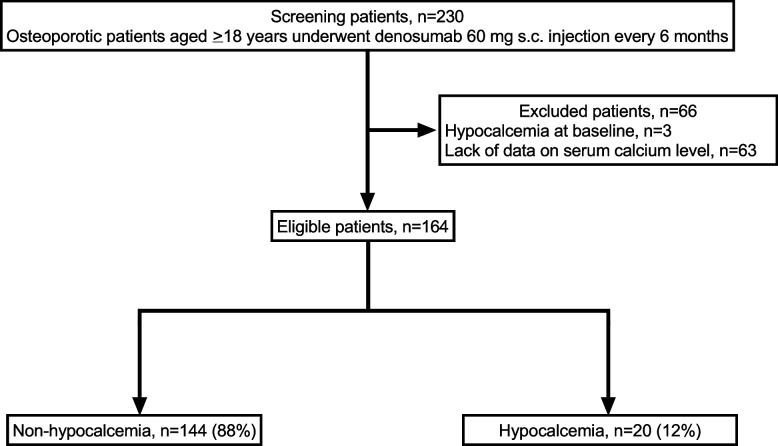
Flow chart of patient selection Abbreviations: s.c.; subcutaneous. Hypocalcaemia was defined as a serum calcium level < 8.5 mg/dL [12]. Serum calcium level was corrected when serum albumin level < 4.0 g/dL [13]. $\text{Corrected calcium}=\text{serum calcium}+\left(4.0-\text{serum albumin}\right)$

The patient selection flow is illustrated in Fig. 1. A total of 230 patients met the inclusion criteria. The dataset comprised 164 patients after excluding hypocalcaemia at baseline (*n* = 3) and no measurement of serum calcium levels after denosumab administration (*n* = 63). A total of 20 (12%) patients developed hypocalcaemia.

A summary of the patient characteristics at the initiation of denosumab is listed in Table 2. The median patient age was approximately 70 years in both groups. Corrected calcium level was significantly lower in the hypocalcaemia than in the non-hypocalcaemia group (non-hypocalcaemia: 9.5 [9.3 to 9.8] mg/dL vs. hypocalcaemia: 9.1 [9.0 to 9.4] mg/dL, *p* < 0.001). For calcium and vitamin D supplementation, the patients consumed the standard dose of calcium carbonate of 1522 mg (as calcium: 610 mg) and cholecalciferol (400 IU) (Denotas®, Daiichi Sankyo, Tokyo, Japan). The number of co-administered loop diuretics was 8 (6%) for the non-hypocalcaemia group and 3 (15%) for the hypocalcaemia group (*p* = 0.114). The furosemide equivalent dose was ≤ 20 mg/day (*n* = 10) and > 20 mg/day (*n* = 1) under oral administration. The date of measuring serum calcium level was not significantly different between groups (non-hypocalcaemia: 183 [70 to 470] days vs. hypocalcaemia: 106 [36 to 296] days, *p* = 0.127).

**Table 2:** Summary of patient characteristics in a retrospective cohort data

	**Non-hypocalcaemia** ***n*** = 144	**Hypocalcaemia** ***n*** = 20	***P*** value
**Demographical data**			
Male, n (%)	12 (8)	4 (20)	0.100
Age, years	71 [63 to 77]	74 [66 to 77]	0.488
Body weight, kg	47 [41 to 55]	45 [41 to 53]	0.385
Body mass index, kg/m^2^	21 [19 to 24]	21 [18 to 22]	0.185
**Co-existing diseases**			
Rheumatoid arthritis, n (%)	24 (17)	2 (10)	0.444
Systemic lupus erythematosus, n (%)	11 (8)	1 (5)	0.671
Vasculitis, n (%)	5 (3)	0 (0)	0.397
Interstitial pneumonia, n (%)	8 (6)	2 (10)	0.436
Inflammatory bowel disease, n (%)	12 (8)	1 (5)	0.605
**Laboratory data**			
Serum Albumin, g/dL	4.1 [3.8 to 4.3]	4.0 [3.6 to 4.1]	0.032
Serum creatinine, mg/dL	0.66 [0.56 to 0.78]	0.74 [0.56 to 1.06]	0.175
eGFR^a^, mL/min/1.73m^2^	69.3 [58.0 to 85.5]	60.2 [43.8 to 93.3]	0.298
eGFR category, n (%)			0.088
≥ 90 mL/min/1.73m^2^	25 (17)	5 (25)	
60 to 89 mL/min/1.73m^2^	79 (55)	5 (25)	
30 to 59 mL/min/1.73m^2^	35 (24)	9 (45)	
15 to 29 mL/min/1.73m^2^	3 (2)	0 (0)	
< 15 mL/min/1.73m^2^	2 (1)	1 (5)	
Serum calcium^b^, mg/dL	9.5 [9.3 to 9.8]	9.1 [9.0 to 9.4]	< 0.001
**Co-administered medication**			
Calcium and vitamin D supplements^c^, n (%)	81 (56)	7 (35)	0.074
Vitamin D preparation, n (%)	46 (32)	7 (35)	0.784
Calcium preparation, n (%)	0 (0)	2 (10)	0.596
Proton pump inhibitors, n (%)	61 (42)	13 (65)	0.057
Loop diuretics, n (%)	8 (6)	3 (15)	0.114
Thiazide diuretics, n (%)	0 (0)	3 (15)	0.515

Data are median [interquartile range] or numbers (%). Continuous data are analyzed with Mann–Whitney U-test. Categorical data are evaluated by Chi-square test. Hypocalcaemia was defined as a serum calcium level < 8.5 mg/dL [12]

*Abbreviations:* *eGFR* estimated glomerular filtration rate

^a^eGFR was estimated using the following Equation [15]

$$eGRF\;=194\times serum\;creatinine^{-1.094}\times age^{-0.287}\times0.739\;\left(if\;female\right)$$

^b^Serum calcium level was corrected when serum albumin level <4.0 g/dL [13]

$$\mathrm{Corrected}\;\mathrm{calcium}\;=\;\mathrm{serum}\;\mathrm{calcium}\;+\;(4.0\;-\;\mathrm{serum}\;\mathrm{albumin})$$

^c^Denotas^®^ (Daiichi Sankyo, Tokyo, Japan)

### A retrospective data analysis

After multivariate logistic regression analysis, the final model identified that denosumab-induced hypocalcaemia was significantly associated with corrected calcium at baseline (OR = 0.032, 95% CI: 0.005 to 0.209, *p* < 0.0001), intake of calcium and vitamin D supplements (OR = 0.285, 95% CI: 0.094 to 0.868, *p* = 0.0270), and administration of loop diuretics (OR = 6.410, 95% CI: 1.005 to 40.90, *p* = 0.0494) (Table 3, AIC = 106.8, r = 0.47, *p* < 0.0001). Interactions were not significant in terms of corrected calcium and loop diuretics (*p* = 0.2884) and calcium and vitamin D supplements and loop diuretics (*p* = 0.8063).

**Table 3:** Multivariate logistic regression analysis of denosumab-induced hypocalcaemia

	**OR**	**95% CI**	***P*** value
Body mass index, per kg/m^2^	0.848	0.701 to 1.024	0.0870
eGFR^a^, per mL/min/1.73m^2^	0.992	0.972 to 1.012	0.4166
Corrected calcium^b^, per mg/dL	0.032	0.005 to 0.209	< 0.0001
Calcium and vitamin D supplements^c^, yes	0.285	0.094 to 0.868	0.0270
Loop diuretics, yes	6.410	1.005 to 40.90	0.0494

Hypocalcaemia was defined as a serum calcium level < 8.5 mg/dL [12]

*Abbreviations:* *OR* Odds ratio, *95% CI* 95% confidence interval, *eGFR* estimated glomerular filtration rate

^a^eGFR was estimated using the following Equation [15]

$$eGRF\;=194\times serum\;creatinine^{-1.094}\times age^{-0.287}\times0.739\;\left(if\;female\right)$$

^b^Serum calcium level was corrected when serum albumin level <4.0 g/dL [13]

$$\mathrm{Corrected}\;\mathrm{calcium}\;=\;\mathrm{serum}\;\mathrm{calcium}\;+\;(4.0\;-\;\mathrm{serum}\;\mathrm{albumin})$$

^c^Denotas^®^ (Daiichi Sankyo, Tokyo, Japan)

**Fig. 2:**
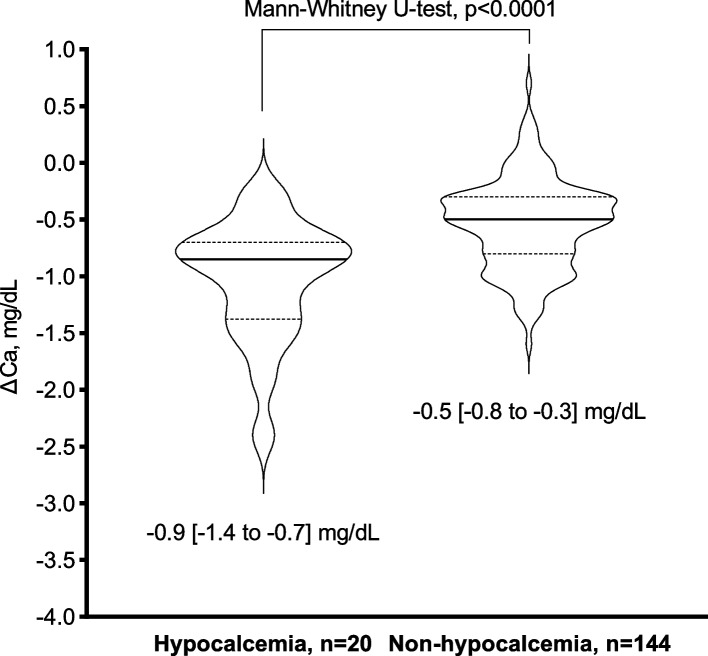
Violin plot of differences in corrected calcium level in patients with and without hypocalcaemia Abbreviations: Ca; serum calcium X-axis and Y-axis represent categories and ΔCa levels, respectively. Solid and dotted lines are median and interquartile range. A difference in serum calcium level (ΔCa, mg/dL) between baseline and nadir was estimated by the equation. $\triangle\mathrm{Ca}=\mathrm{serum}\;\mathrm{calcium}\;\mathrm{at}\;\mathrm{nadir}-\mathrm{serum}\;\mathrm{calcium}\;\mathrm{at}\;\mathrm{baseline}$ . Hypocalcaemia was defined as a serum calcium level < 8.5 mg/dL [12]. Serum calcium level was corrected when serum albumin level < 4.0 g/dL [13]. $\text{Corrected calcium}=\text{serum calcium}+\left(4.0-\text{serum albumin}\right)$

The trends in corrected calcium are illustrated in Supplementary Materials 1. There was a significant decrease in ΔCa in hypocalcaemia than in non-hypocalcaemia (hypocalcaemia: -0.9 [-1.4 to -0.7] mg/dL vs. non-hypocalcaemia: -0.5 [-0.8 to -0.3] mg/dL, *p* < 0.0001, Fig. 2). The hypocalcaemia group showed no difference in ΔCa (Dmab + LD, *n* = 3: -1.0 [-1.2 to -0.8] mg/dL vs. Dmab alone, *n* = 17: -0.8 [-1.5 to -0.7] mg/dL, *p* = 0.7904, Supplementary Materials 2). However, in the non-hypocalcaemia group, ΔCa was significantly lower in Dmab + LD than in Dmab alone (Dmab + LD, *n* = 8: -0.9 [-1.3 to -0.7] mg/dL vs. Dmab alone, *n* = 136: -0.5 [-0.8 to -0.3] mg/dL, *p* = 0.0156).

## Discussion

We found that co-administration of loop diuretics was a risk factor for denosumab-induced hypocalcaemia in patients with osteoporosis. However, the risk of hypocalcaemia was significantly lower when serum calcium levels were higher at the initiation of denosumab or when calcium and vitamin D supplements were co-administered. The influence of loop diuretics on denosumab-induced hypocalcaemia was evident even in patients without hypocalcaemia. We believe that these findings are valuable for the early detection of denosumab-induced hypocalcaemia and for avoiding lethal events such as cardiac arrhythmia.

The incidence of denosumab-induced hypocalcaemia was consistent with previously reported findings [6, 18, 19]. Real-world data have demonstrated that hypocalcaemia occurs several weeks to more than two years after the first administration of denosumab [4]. In contrast, a long-term extension of a randomized controlled trial revealed that hypocalcaemia occurred at a rate of less than 0.1 per 100 patient-years for up to 10 years [20]. The difference observed in the incidence may attributed to the amount of calcium supplementation. All participants registered in the randomized trial orally received 1,000 mg of calcium (vs. 610 mg of calcium as a usual dose of Denotas®) [2, 20]. It remains critical to closely monitor serum calcium levels during the long-term administration of denosumab based on the factors affecting denosumab-induced hypocalcaemia in clinical practice.

Our finding detected no impact of kidney function on the risk of hypocalcaemia. However, eGFR is a risk factor for denosumab-induced hypocalcaemia [4, 21]. In particular, a previous study demonstrated an increased risk in the development of hypocalcaemia in patients who had eGFR < 30 mL/min/1.73m^2 ^[22]. The present study included a limited number of patients who had a severe stage of chronic kidney disease. Therefore, it is impossible to detect eGFR as a risk factor for denosumab-induced hypocalcaemia. Moreover, co-administration of proton pump inhibitors impairs mineral metabolism by suppressing calcium absorption in the gut [23]. Although the clinical study demonstrated that co-administration of proton pump inhibitors is a risk factor for the denosumab-induced hypocalcaemia [24], our finding detected no significant relationship between denosumab-induced hypocalcaemia and proton pump inhibitors. We cannot offer a strong conclusion, but additional studies should be conducted to clarify new findings.

The present study revealed that a high baseline serum calcium level is a protective factor against denosumab-induced hypocalcaemia. Although approximately 90% of patients administered calcium and vitamin D supplementation [22], our finding was confirmed in the dataset where only 35% of hypocalcaemic patients received calcium and vitamin D supplementation, which prevents the generalization of findings. We could not obtain a clear conclusion about this reason in this study. However, an omission of introducing medications without valid reasons (under-prescription) can be problematic in the elderly [25]. We speculate that under-prescription of calcium and vitamin D supplementation was observed when given denosumab within the normal range of calcium levels. The development of hypocalcaemia is reportedly more common when patients have lower baseline serum calcium levels [4, 6], which is consistent with our findings. Adding calcium and vitamin D supplements to denosumab prescription ameliorates the calcium-lowering effects of denosumab and consistently increases bone mineral density [19]. Since calcium and vitamin D supplementation are key factors in enhancing the net benefit-risk balance of denosumab, physicians should consider co-prescribing calcium and vitamin D supplements while administering denosumab unless contraindicated.

We observed an increased risk (or positive signal) of hypocalcaemia when simultaneously given denosumab and loop diuretics (Tables 1 and 3). Our multivariate model did not have any interaction of loop diuretics with significant variables. The potential for drug-drug interactions in denosumab-induced hypocalcaemia has rarely been investigated [26]. A previous study reported a trend toward an increased risk of denosumab-induced hypocalcaemia while receiving loop diuretics [8]. A case series study reported that denosumab-induced hypocalcaemia occurred in some patients taking hypocalcaemic diuretics, such as furosemide [27]. Importantly, calcium homeostasis was affected differently depending on the furosemide dose, with the data indicating that a daily dose of furosemide > 60 mg may decrease the serum calcium concentration through accelerated urinary excretion [28]. Nonetheless, co-administration of loop diuretics was a significant variable for denosumab-induced hypocalcaemia, although nearly all of the study patients received furosemide ≤ 20 mg. Based on these findings, we recommend that consecutive administration of denosumab should be performed with particular attention to hypocalcaemia even in patients given a lower dose of loop diuretics.

Co-administered loop diuretics significantly lowered corrected calcium levels in patients with non-hypocalcaemia but not in those with hypocalcaemia (Supplementary Material 2). A basic experiment found that the calcium-lowering effect of loop diuretics was attenuated after long-term administration through a compensatory mechanism of intestinal calcium absorption in proportion to activated vitamin D levels [29]. Therefore, adequate calcium supplementation is important to compensate for this calcium-lowering effect. It should also be noted that loop diuretics inhibit calcium reabsorption, increasing urinary calcium excretion in secondary hyperparathyroidism [9]. We speculate that a synergistic effect of combination therapy with denosumab and loop diuretics disturbs bone resorption and urinary calcium excretion, resulting in a decrease in serum calcium levels.

This study had some limitations that must be considered to interpret the findings correctly. First, this was a retrospective observational study; therefore, there is a possibility of unknown bias and confounding factors. Second, collecting data on calcium intake and laboratory parameters such as ionized calcium levels, bone turnover markers, and 1,25-dihydroxy vitamin D and parathyroid hormone levels was impossible. Third, it was challenging to generalize the results to cancer patients treated with denosumab. Fourth, it was difficult to assess medication adherence and the causality of hypocalcaemia. Fifth, our findings require further verification among those receiving a high dose of furosemide. Sixth, we did not utilize propensity score matching because of a limited number of patients who received loop diuretics. Finally, we could not analyze hypocalcaemia caused due to hypomagnesemia after the administration of loop diuretics [30].

In conclusion, loop diuretics should be considered as a risk factor under denosumab administration. A close monitoring of serum calcium level and patient education about hypocalcaemia will be a valuable approach during the administration of denosumab with loop diuretics. Optimal calcium concentrations and calcium and vitamin D supplementation can substantially reduce the burden of denosumab-induced hypocalcaemia. Further study is required to identify the effect of thiazide diuretics on the risk of denosumab-induced hypocalcaemia.

## Supplementary Information

Supplementary Material 1.

## Data Availability

The data that support the findings of this study are available on request from the corresponding author.

## Abbreviations

eGFR: Estimated glomerular filtration rate
JADER: Japanese Adverse Drug Event Report database
MedDRA/J: Medical Dictionary for Regulatory Activities
ROR: Reporting odds ratio
95% CI: 95% Confidence interval
ΔCa: Difference in corrected calcium level
AIC: Akaike's Information Criterion
OR: Odds ratio
Dmab + LD: Denosumab plus loop diuretics
Dmab alone: Denosumab alone
