# Supplementary material for: Influence of loop diuretics on denosumab-induced hypocalcaemia in osteoporosis: a retrospective observational analysis
Source: J Pharm Health Care Sci. 2024 Sep 27;10:60. doi: 10.1186/s40780-024-00380-8 (PMC11437979; doi:10.1186/s40780-024-00380-8)
Supplement: Supplementary file 1 — Supplementary Material 1. [file 40780_2024_380_MOESM1_ESM.docx]

**Supplementary Material 1 Trends in corrected calcium level according to loop diuretics and hypocalcemic events**

|  | **Baseline** | **Nadir** | **P value** |
| --- | --- | --- | --- |
| **Hypocalcaemia** |  |  |  |
| All, n=20 | 9.1 [9.0 to 9.4] | 8.3 [8.2 to 8.4] | <0.0001 |
| Denosumab + loop diuretics, n=3 | 9.4 [9.0 to 9.4] | 8.2 [8.2 to 8.4] | 0.2500 |
| Denosumab alone, n=17 | 9.1 [8.9 to 9.4] | 8.4 [8.1 to 8.4] | <0.0001 |
| **Non-hypocalcaemia** |  |  |  |
| All, n=144 | 9.5 [9.3 to 9.8] | 9.0 [8.8 to 9.2] | <0.0001 |
| Denosumab + loop diuretics, n=8 | 9.9 [9.7 to 10.0] | 9.0 [8.8 to 9.2] | 0.0156 |
| Denosumab alone, n=136 | 9.5 [9.3 to 9.8] | 9.0 [8.8 to 9.2] | <0.0001 |

Data are median [interquartile range] and compared by Wilcoxon signed-rank test. Hypocalcaemia was defined as a serum calcium level <8.5 mg/dL ^12)^. Serum calcium level was corrected when serum albumin level <4.0 g/dL ^13)^.

$$Corrected calcium=serum calcium+\left( 4.0-serum albumin \right)$$

**Supplementary Material 2 Violin plot of differences in serum calcium level in patients with and without loop diuretics considering hypocalcaemia**

Abbreviations: Ca; serum calcium

X-axis and Y-axis represent categories and ΔCa levels, respectively. Solid and dotted lines are median and interquartile range. Opened and gray violins are data on groups without and with loop diuretics, respectively. A difference in serum calcium level (ΔCa, mg/dL) between baseline and nadir was estimated by the equation.

$$\Delta Ca=serum calcium at nadir-serum calcium at baseline$$

Hypocalcaemia was defined as a serum calcium level <8.5 mg/dL ^12)^. Serum calcium level was corrected when serum albumin level <4.0 g/dL ^13)^.

$$Corrected calcium=serum calcium+\left( 4.0-serum albumin \right)$$
